# Prostanoids and Resolution of Inflammation – Beyond the Lipid-Mediator Class Switch

**DOI:** 10.3389/fimmu.2021.714042

**Published:** 2021-07-12

**Authors:** Tobias Schmid, Bernhard Brüne

**Affiliations:** ^1^ Institute of Biochemistry I, Faculty of Medicine, Goethe-University Frankfurt, Frankfurt, Germany; ^2^ German Cancer Consortium (DKTK) Partner Site Frankfurt, Frankfurt, Germany; ^3^ Frankfurt Cancer Institute, Goethe-University Frankfurt, Frankfurt, Germany; ^4^ Fraunhofer Institute for Translational Medicine and Pharmacology ITMP, Frankfurt, Germany

**Keywords:** prostaglandin, prostacyclin, thromboxane, specialized pro-resolving mediator, inflammation, resolution, macrophage

## Abstract

Bioactive lipid mediators play a major role in regulating inflammatory processes. Herein, early pro-inflammatory phases are characterized and regulated by prostanoids and leukotrienes, whereas specialized pro-resolving mediators (SPM), including lipoxins, resolvins, protectins, and maresins, dominate during the resolution phase. While pro-inflammatory properties of prostanoids have been studied extensively, their impact on later phases of the inflammatory process has been attributed mainly to their ability to initiate the lipid-mediator class switch towards SPM. Yet, there is accumulating evidence that prostanoids directly contribute to the resolution of inflammation and return to homeostasis. In this mini review, we summarize the current knowledge of the resolution-regulatory properties of prostanoids and discuss potential implications for anti-inflammatory, prostanoid-targeted therapeutic interventions.

## Introduction

Inflammation is an integral part of protective host responses against pathogens or injury (1). Herein, inflammatory processes usually follow a defined sequence of reactions characterized by the rapid induction of a pro-inflammatory response, which is closely followed by an anti-inflammatory response. To regain homeostasis resolution of inflammation represents an integral and crucial part of acute inflammation. In fact, failure to completely resolve inflammatory processes is associated with the emergence of chronic inflammatory conditions. While resolution was considered to merely represent the downregulation or inactivation of inflammatory mediators for a long time, it is now appreciated to be active and complex, involving the formation of pro-resolving mediators (2). Moreover, it is widely accepted that resolution processes are initiated early during inflammation, and classical pro-inflammatory mediators have been shown to directly impact on resolution as well (3). Therefore, pro-resolving therapeutic approaches are increasingly being considered rather than anti-inflammatory ones as the latter might diminish the host response against pathogenic challenges (4, 5). Since lipid mediators play a crucial, yet often ambivalent role during the inflammatory process, and prostanoid synthesis represents a major target for anti-inflammatory therapies, the present mini review aims to recapitulate the current understanding of the role of prostanoids in the context of resolution of inflammation.

## The Course of Inflammation

### Cellular Mediators

Upon an insult (e.g., pathogen contact, injury) local resident immune cells are the first responders. Among these, tissue resident macrophages (MΦ) represent not only an important first line of defense but even more importantly they establish an environment prone to recruit neutrophils and monocytes from the circulation. Neutrophils are main executers in the acute inflammatory environment, i.e. they produce high amounts of reactive oxygen and nitrogen species to eliminate pathogens, and secrete a plethora of inflammatory mediators (6). Within the inflammatory environment neutrophils rapidly die by apoptotic processes and are phagocytosed by MΦ in a process termed efferocytosis (7, 8). Importantly, uptake of apoptotic cells (AC) induces a shift in MΦ polarization from a pro-inflammatory to an anti-inflammatory and immunomodulatory phenotype (9–14). MΦ and other antigen-presenting cells such as dendritic cells (DC) eventually activate adaptive immune responses, which ensures complete removal of the invading pathogens and enables accelerated responses upon anew contact to the triggering stimulus (15). The return to cellular homeostasis further requires successful emigration of infiltrated immune cells from the cleared site of inflammation (16). In contrast to the concept of a rapid return to homeostasis, recent reports provided evidence that resolution is followed by a longer lasting, immune-suppressive post-resolution phase characterized by infiltrating regulatory T cells (Treg) and myeloid-derived suppressor cells (MDSC), but also MΦ, which might be decisive for a successful adaptation vs. chronic inflammation and/or autoimmunity (17–19).

### Lipid Mediators

**Figure 1:**
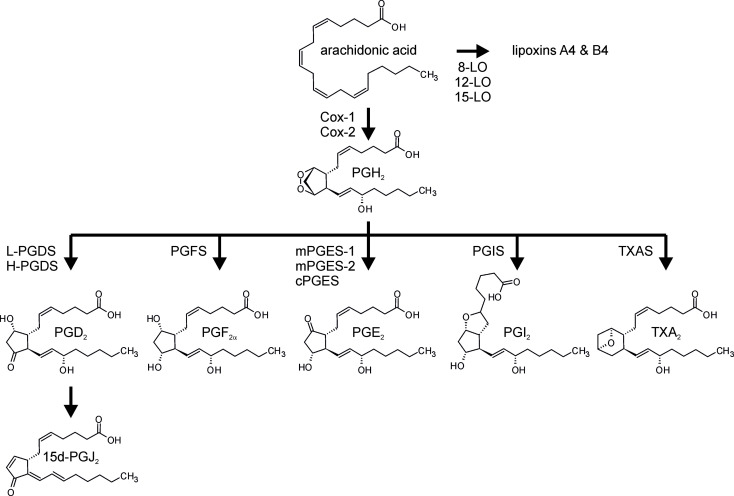
Prostanoid synthesis. Arachidonic acid, liberated from membrane phospholipids by phospholipase A_2_, is converted to prostaglandin H_2_ (PGH_2_) by the dual peroxidase/cyclooxygenase activity of the cyclooxygenases (Cox-1, Cox-2). PGH_2_ serves as the substrate for the terminal synthases to produce PGD_2_, PGE_2_, PGF_2α_, PGI_2_, and thromboxane A_2_ (TXA_2_). PGD_2_ is further dehydrated and isomerized spontaneously to 15-deoxy−Δ^12,14^-PGJ_2_ (15d-PGJ_2_). Alternatively, arachidonic acid can be converted to lipoxins, i.e. SPM, *via the* activity of the lipoxygenases (LO). L-PGDS, lipocalin-type PGD synthase; H-PGDS, hematopoietic-type PGDS; mPGES, microsomal PGES; cPGES, cytosolic PGES; TXAS, TXA synthase.

Regulation and execution of inflammatory reactions is mediated by soluble mediators, such as cytokines and chemokines. In addition, bioactive lipids emerged as crucial factors during all phases of the inflammatory process (20, 21). Specifically, while leukotrienes and prostaglandins appear early during the onset of inflammation, specialized pro-resolving mediators (SPM), such as lipoxins, resolvins, and maresins, are produced later on, facilitating the resolution of inflammation (22). Prostanoids, like leukotrienes, belong to the eicosanoid family of lipid mediators (23). Both classes are synthesized from arachidonic acid (AA) after the latter is released from membrane phospholipids by phospholipase A_2_ (24). While leukotrienes are synthesized by the lipoxygenases, prostanoid formation initially requires conversion of AA to the unstable prostaglandin H_2_ (PGH_2_) *via* the bifunctional cyclooxygenases 1 (Cox-1) and 2 (25, 26). In line with the established pro-inflammatory function of the prostanoids, the cyclooxgenases became an important target in the therapy of inflammatory diseases, and as of today, non-steroidal anti-inflammatory drugs (NSAIDs) rank amongst the most important anti-inflammatory drugs (27, 28). Of note, while Cox-1 is constitutively expressed in most cells, Cox-2 often is inducible and emerged as the more important cyclooxygenase in the context of inflammation. Consequently Cox inhibitors (Coxibs) selectively targeting Cox-2 emerged as promising novel anti-inflammatory therapeutics (29, 30). The short-lived Cox product PGH_2_ is then substrate to specific synthases, which produce potent prostanoids including PGD_2_, PGE_2_, PGF_2α_, and PGI_2_, as well as thromboxane A_2_ (TXA_2_) (**Figure 1**).

Interestingly, the production of the different lipid mediators appears to be tightly connected across the course of inflammation. For example, the presumably pro-inflammatory PGE_2_ was shown to attenuate the synthesis of leukotrienes and to induce the production of SPM, thus initiating the so-called lipid-mediator class switch (31). While the SPM-regulatory impact of prostanoids on the resolution of inflammation is rather well characterized, there is accumulating evidence that prostanoids also directly impinge on other aspects of the resolution process. In the following sections, we will therefore summarize the current understanding of the role of the most prominent prostanoids in resolution of inflammation, aside from the aforementioned lipid-mediator class switch, with a special focus on PGE_2_.

## Prostanoids in the Resolution of Inflammation

### Thromboxane A_2_

TXA_2_ is predominantly produced by platelets, but also at appreciable amounts by MΦ (32). Interestingly, while thromboxane A synthase (TXAS) appears to be coupled to the activity of Cox-1 and Cox-2 constitutes the dominant Cox in inflammatory conditions, the activity of TXA_2_ is largely pro-inflammatory (33, 34). In fact, there is little evidence that TXA_2_ might contribute to the resolution of inflammation. In line, Kupffer cell-derived TXA_2_ was shown to contribute to liver fibrosis, a common outcome of ineffective resolution (35). The observation that TXA_2_ synthesis is induced by pro-inflammatory stimuli, while it is suppressed upon inflammatory restimulation (36), further suggest that the prevention of TXA_2_, e.g. *via* redirection of Cox-1-provided PGH_2_ towards PGE_2_ synthesis, might be part of the immune-suppressive environment typical for the post-resolution phase (18).

### Prostaglandin I_2_ (Prostacyclin)

PGI_2_ (prostacyclin) has been characterized as the counterpart of TXA_2_ as it inhibits platelet aggregation and acts as a potent vaso- and bronchodilator (37). It is produced primarily by vascular endothelial and smooth muscle cells, yet other cells such as fibroblasts and dendritic cells also synthesize PGI_2_ (38). In the context of inflammation, PGI_2_ was shown to inhibit LPS-induced expression of pro-inflammatory cytokines in MΦ, DC, CD4^+^ T cells, and endothelial cells (39–42). PGI_2_ further synergizes with anti-inflammatory cytokines interleukin-4 (IL-4) and IL-13 to suppress pro-inflammatory cytokines (43). Along the same lines, PGI_2_ receptor (IP) deficient mice displayed stronger allergic inflammation, which was attributable to enhanced Th2 cell function (44). Thus, PGI_2_ emerges as a predominant anti-inflammatory mediator, positioning it also as a counterpart of TXA_2_ in the context of inflammation.

### Prostaglandin D_2_

PGD_2_ is produced by numerous immune cells including activated MΦ, DC, Th2 cells, eosinophils, platelets, but also endothelial cells (45). However, since its main source are mast cells (46, 47), PGD_2_ has been characterized extensively in the context of allergic reactions (48). PGD_2_ can further be metabolized to the cyclopentenone, PGJ_2_-type prostanoids, including PGJ_2_ and 15d-PGJ_2_, which also display biological activity in the context of resolution of inflammation (49). PGD_2_ binds to the PGD_2_ receptors 1 (DP1) and DP2 [also known as chemoattractant receptor-homologous molecule expressed on Th2 cells (CRTH2)] (50). Activation of DP2 is of specific importance in allergic inflammation, where mast cell-derived PGD_2_ stimulates the recruitment of innate lymphoid type 2 cells (51) and Th2 cells (52), and causes activation of these as well as of basophils and eosinophils (53). While PGD_2_ was shown to contribute to allergic inflammation, PGD_2_ synthase (PGDS) decreases during the acute inflammatory phase, while it rises again at later stages corresponding to the resolution phase in animal models (54). PGD_2_ and 15d-PGJ_2_ both exert pro-inflammatory functions *via* CRTH2 (55). In contrast, PGD_2_-dependent activation of DP1 as well as 15d-PGJ_2_-mediated activation of peroxisome proliferator-activated receptor γ (PPARγ) inhibit the production of inflammatory cytokines and chemokines by antigen-presenting cells including DC or MΦ by interfering with inflammatory transcription factors nuclear factor kappa B (NFκB), activator protein 1 (AP1), and signal transducer and activator of transcription 3 (STAT3) (56–58) and additionally by enhancing the activity of anti-inflammatory nuclear factor erythroid 2-like 2 (Nrf2) (59, 60). Consequently, PGD_2_ and 15d-PGJ_2_ support emigration of MΦ to the draining lymph nodes and attenuate the recruitment of leukocytes (54). They further inhibit effector functions of and induce apoptosis in T lymphocytes (57). Thus, PGD_2_ contributes to normalize the local environment, an important aspect of the resolution of inflammation (61).

### Prostaglandin F_2α_

PGF_2α_ is produced by the aldo-keto reductase (AKR) 1C3, also known as PGF_2α_ synthase (PGFS) using PGH_2_ or PGD_2_ (62). Alternatively, PGE_2_ can be converted to PGF_2α_ by AKR1C1 or AKR1C2 (63). While PGF_2α_ is synthesized in most tissues (64), its prime site of production is the female reproductive system (65), where PGF_2α_ has been shown to be of functional importance (66, 67). Nevertheless, PGF_2α_ also appears to be involved in inflammatory processes (64, 68). PGF_2α_ is elevated in rheumatoid arthritis (69) and was shown to contribute to and correlate with fibrosis (70, 71), which is characteristic for insufficient resolution. Interestingly though, PGFS expression and concomitantly PGF_2α_ levels decrease similar to PGD_2_ during acute inflammation, only to increase again in the resolution phase (72), which might be indicative for an active role of PGF_2α_ in the resolution of inflammation. Yet, the exact role of PGF_2α_ in inflammation-resolving processes remains to be determined.

### Prostaglandin E_2_

The best characterized and presumably most important prostanoid in the context of inflammation is PGE_2_. It can be synthesized by all cell types. In the context of inflammation the prime producers are fibroblasts, epithelial cells, and immune cells (73). PGE_2_ exerts its functions *via* four G-protein-coupled PGE_2_ receptors (EP1-4) (74). While EP2 and 4 represent Gα_s_-coupled receptors increasing cAMP levels upon activation (75), the Gα_i_-coupled EP3 variants inhibit adenylate cyclase, thus reducing cAMP (76), and Gα_q_ EP1 enhances intracellular Ca^2+^ levels (74). The distinct downstream signals as well as the cell type-specific distribution of the receptors, and their differential sensitivity for PGE_2_ account for the diverse functions of PGE_2_ also in inflammation (77).

**Figure 2:**
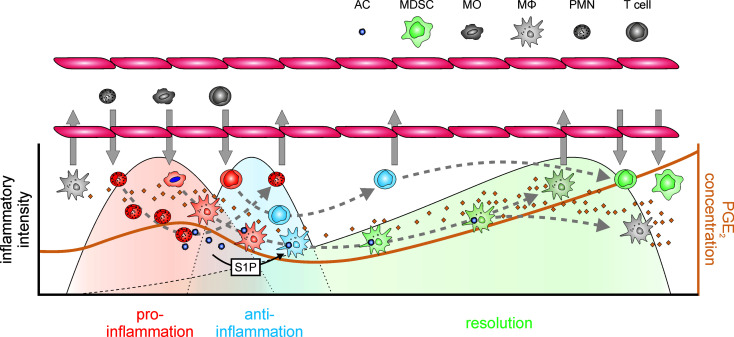
PGE_2_ in the context of inflammation. An inflammatory insult is recognized by resident immune cells such as resident MΦ. Upon inflammatory stimulation Cox-2 and mPGES-1 are induced in MΦ resulting in increased PGE_2_ synthesis. PGE_2_ then contributes to the MΦ-mediated recruitment of neutrophils, which act as a first line of defense to eliminate the pathogenic stimulus and incite further inflammatory responses. Already at this early stage of the inflammatory process PGE_2_ initiates the lipid mediator class switch towards the production of specialized pro-resolving mediators (SPM) including lipoxins, resolvins, maresins, and protectins e.g. in neutrophils. Neutrophils are rapidly followed by monocytes again facilitated by PGE_2_, which upon infiltration into the affected tissue differentiate into pro-inflammatorily activated MΦ and release soluble mediators including cytokines and chemokines, as well as further PGE_2_. PGE_2_-triggered transcriptional programs eventually induce post-transcriptional feedback circuits, which reduce Cox-2 mRNA stability and thus protein expression to eventually inhibit PGE_2_ production, and also attenuate the expression of pro-inflammatory cytokines such as TNF. In addition, anti-inflammatory mediators (including IL-10) are induced restricting the intensity of the inflammatory reactions. Within the inflammatory niche, activated neutrophils rapidly undergo apoptotic cell death and are phagocytosed by MΦ, which causes a shift in MΦ polarization towards an alternatively activated, immune-modulatory, resolution phenotype characterized by the secretion of e.g. TGFβ. Apoptotic cells further release sphingosine-1-phosphate (S1P), which enhances the mRNA stability of Cox-2 again, resulting in increasing PGE_2_ production. Within the resolving environment PGE_2_ supports a shift from Th1 T cells to the repair-associated Th2-type further supporting tissue normalization. In addition, PGE_2_ attenuates expression of CX3CL1 in MΦ, thereby eventually allowing their emigration from the resolving tissue. PGE_2_ further supports the recruitment of T cells and myeloid cells, which differentiate into regulatory T cells and myeloid-derived suppressor cells, respectively, thereby establishing an immune-suppressive post-resolution environment. *Lower part*: During inflammation, PGE_2_ levels transiently increase in the acute inflammatory phase. After a decrease during the anti-inflammatory and the early resolution phase, PGE_2_ tends to increase again during the progress of resolution, reaching highest levels in the post-resolution phase. AC, apoptotic cells; MΦ, macrophages (gray – naïve, resident; red – pro-inflammatory; blue – anti-inflammatory; green – resolution phase); MDSC, myeloid-derived suppressor cells; MO, monocytes; PMN, neutrophils (gray – naïve, red – inflammatory); T cells (red – Th1; blue – Th2; green – Treg); ⇨, infiltration/emigration; →, development within the inflammatory niche.

Upon inflammatory stimulation, both the expression of Cox-2 and microsomal PGE_2_ synthase 1 (mPGES-1) are induced in MΦ (78), skewing the prostanoid spectrum towards PGE_2_ production in the acute inflammatory phase (73). PGE_2_ signals towards enhanced recruitment of neutrophils, MΦ, and mast cells (79–81) and enhances the expression and secretion of pro-inflammatory cytokines in DC, MΦ, and T cells (82–84). Its NFκB-activating properties further support neutrophil survival, thereby extending the pro-inflammatory impact of neutrophils in the inflammatory niche (85, 86). Yet, this initial increase in PGE_2_ is only moderate and transient in character, and PGE_2_ levels rise again during the resolution phase, eventually increasing to much higher levels in the post-resolution phase (18, 87, 88). This seemingly biphasic regulation of PGE_2_ might at least in part be due to a shift from transcriptional to post-transcriptional programs governing not only PGE_2_ production, but more generally the course of inflammation (89). With respect to the regulation of PGE_2_ synthesis, Cox-2 rapidly accumulates during early inflammation. In a negative feedback loop, elevated PGE_2_ induces the expression of dual specificity phosphatase 1 (DUSP1), thereby enhancing the activity of the RNA-binding protein tristetraprolin (TTP), which destabilizes the mRNA of Cox-2, but also of pro-inflammatory tumor necrosis factor (TNF) (90). The second wave of PGE_2_ production by MΦ appears to be facilitated by another increase of Cox-2 expression induced by sphingosine-1-phosphate released from apoptotic cells, which activates the RNA-stabilizing protein human antigen R (HuR) in MΦ to increase Cox-2 expression (91). As a side note, while PGE_2_ is predominantly produced in a Cox-2/mPGES-1 dependent manner during the inflammatory and early resolution phase, PGE_2_ levels during the post-resolution phase are approx. 3-fold higher, which is due to enhanced Cox-1/mPGES-1 expression in MΦ (18). While these findings might explain the kinetics of PGE_2_ production and even some of the inhibitory effects of PGE_2_ on pro-inflammatory mediators, further evidence for an immunosuppressive function of MΦ-derived PGE_2_ emerged in the tumor context, where PGE_2_ inhibits CD80 expression *via* EP2, thereby attenuating activation of cytotoxic T cells (92, 93). Similarly, PGE_2_ suppresses cytolytic functions of NK cells (94, 95) and inhibits phagocytic and bacteria killing activities of MΦ, thus preventing the establishment of appropriate inflammatory, anti-microbial responses largely *via* EP2-dependent cAMP induction (96–98). In line, EP2- and EP4-signaling limits secretion of TNF and IL-1β, and enhances expression of anti-inflammatory IL-10 in response to LPS by microglia (99), i.e. resident MΦ of the central nervous system (100). In general, EP2- and EP4-activation by PGE_2_ appears to be crucial to establish an anti-inflammatory and resolving MΦ phenotype (101, 102), which is characterized by further immune-modulatory factors such as transforming growth factor β (TGFβ) (103). Nevertheless, there are contradictory reports regarding the exact impact of PGE_2_ on T cell functions. On the one hand, PGE_2_ appears to inhibit IL-2 production by T cells, thereby attenuating both T cell activation and activation-dependent apoptosis (104–106). On the other hand, while PGE_2_ appears to contribute to sustained inflammation by differentiation and activation of Th1 and γδ T cells, considered to support inflammation (107–109), other findings indicate that PGE_2_ selectively inhibits Th1 cytokine production leaving Th2 cytokines, such as IL-4 and IL-5, unaffected (110, 111), thus provoking a PGE_2_-dependent shift towards Th2 responses, which are associated with repair mechanisms instead (112–114). This notion is substantiated by the high levels of PGE_2_ observed in Th2-driven diseases such as atopic allergy (115). Indeed, the intricate impact of PGE_2_ on the balance between different T cell subtypes might be one of the key mechanisms how PGE_2_ affects a self-limiting inflammation throughout resolution and post-resolution phases. Elevated PGE_2_ impairs interferon γ (IFN γ) synthesis, thereby directly attenuating Th1 responses, while leaving Th2 responses unaltered (110, 111). PGE_2_ further favors Th17 responses *via* EP2 and EP4 by shifting the IL-12/IL-23 balance towards Th17-supportive IL-23 (116–118). While Th17 cells are largely inflammatory in nature contributing to severe inflammatory diseases (107, 119), they have been shown to be highly plastic, being able to trans-differentiate into Treg thereby contributing to resolution of inflammation (120). Yet, PGE_2_ not only promotes differentiation of naïve T cells or Th17 cells towards Tregs (121, 122), it also supports further expansion of differentiated Tregs (123). Conclusively, the concentration, source, and timing of PGE_2_ appear critical to determine the exact T cell response in the context of inflammatory responses. Moreover, while the pro-resolving activity of cyclooxygenase metabolites has long been attributed predominantly to PGD_2_ and 15d-PGJ_2_ (124), PGE_2_ emerged as an important facilitator of the lipid-mediator class switch inducing not only the production of PGD_2_ and its derivatives, but also of the so-called specialized pro-resolving mediators (SPM) (20). SPM, synthesized by 15-lipoxygenase (ALOX15) (31, 125–127), are key players in the resolution of inflammation (114, 128). PGE_2_ induces the expression of the relevant lipoxygenases, thereby skewing the balance towards a pro-resolving lipid mediator profile (129, 130). While SPM levels are mostly considered to reciprocally reflect PGE_2_ levels during the course of inflammation, they in fact coexist (131). The exact temporal and spatial distribution of both PGE_2_ and SPM might eventually determine the course of the resolution of inflammation. Of note, SPM also have been shown to affect the T cell balance (132–134). Yet, there is mounting evidence that PGE_2_ also directly supports further resolution-associated functions. Along these lines, PGE_2_ has been shown to inhibit pro-inflammatory cytokine production (99, 135) and to stimulate the expression of anti-inflammatory cytokines (136, 137), thereby contributing to the early steps of the resolution process (138). Furthermore, blocking PGE_2_ synthesis attenuated efficient resolution in a peritonitis model by preventing the emigration of MΦ from the site of inflammation in a CX3CL1-dependent manner (87). Extending beyond its impact on the resolution phase, PGE_2_ contributes to the immune suppressive post-resolution phase, where PGE_2_ suppresses innate immune responses, inhibits lymphocyte functions, and contributes to the generation and activity of Treg (121, 139) and MDSC (140). While these immune suppressive effects appear negative in the context of novel infections, they lower the risk of autoimmune responses (18, 19) (**Figure 2**). The complexity of the resolution of inflammation both at cellular as well as (lipid) mediator level, highlights the need for further studies unraveling details of the resolution phase to allow for the development of refined therapeutic intervention strategies, especially for chronic inflammatory diseases lacking proper resolution mechanisms.

## Therapeutic Considerations

Cox inhibitors are amongst the most widely used over the counter anti-inflammatory drugs worldwide (141). Despite the undisputed beneficial effects of the broad spectrum Cox inhibitors, specific Cox-2 inhibitors (Coxibs) were developed to more selectively interfere with the production of inflammation-associated prostanoids (142). Due to the prominent role of PGE_2_ in the establishment of inflammatory processes, recent therapeutic approaches aimed at inhibiting the inflammation-associated terminal PGE_2_ synthase mPGES-1 to selectively block the production of PGE_2_ only (73, 143, 144). Yet, considering the major impact of PGE_2_ on successful resolution of inflammation, therapeutic approaches targeting PGE_2_ synthesis should be critically revisited as continued PGE_2_ inhibition in inflammatory diseases might be expected to lead to chronic diseases due to insufficient resolution. In fact, even attenuating inflammation itself might interfere with successful resolution, since the process of resolution is initiated already very early during the inflammatory process (3). Thus, it might be warranted to focus on therapeutic approaches promoting resolution rather than on anti-inflammatory ones in the future (4, 145). Indeed, there are numerous efforts to target SPM production or receptors (146). Considering promising effects of PGE_2_ receptor antagonists (e.g. EP4) in the context of chronic inflammatory diseases (147), it will be interesting to see combinatorial approaches in the future.

## Author Contributions

TS and BB wrote and edited the manuscript. All authors contributed to the article and approved the submitted version.

## Funding

This work was supported by grants of the DFG (GRK 2336 TP6, SCHM2663/7, SFB1039 B04).

## Conflict of Interest

The authors declare that the research was conducted in the absence of any commercial or financial relationships that could be construed as a potential conflict of interest.
